# 2-[2,6-Bis(pyrazin-2-yl)pyridin-4-yl]benzoic acid

**DOI:** 10.1107/S1600536814008496

**Published:** 2014-04-18

**Authors:** Ying Shuai, Xiang-Yang Wang, Jing-Wei Dai, Jian-Zhong Wu

**Affiliations:** a School of Chemistry and Environment, South China Normal University, Guangzhou 510006, People’s Republic of China

## Abstract

In the title compound, C_20_H_13_N_5_O_2_, the two pyrazine rings are nearly coplanar with the central pyridine ring, forming dihedral angles of 2.21 (9) and 4.57 (9)°. In contrast, the strong steric hindrance caused by the *ortho*-carboxyl group on the phenyl ring makes this ring rotate out of the attached pyridine ring plane by 52.60 (9)°. The carboxyl group is twisted from the phenyl ring by 22.6 (1)°. In the crystal, aromatic π–π stacking inter­actions [centroid–centroid distances = 3.9186 (4) and 3.9794 (5) Å] occur between the anti­parallel mol­ecules, generating infinite chains along [100]. O—H⋯O hydrogen bonds connect the chains, leading to the formation of a two-dimensional supra­molecular network parallel to (010). Inter­molecular C—H⋯N hydrogen bonds are also observed.

## Related literature   

For background to terpyridine compounds, see: Constable (2008 ▶); Eryazici *et al.* (2008 ▶); Schubert *et al.* (2006 ▶); Wild *et al.* (2011 ▶); Zadykowicz & Potvin (1999 ▶); Wang & Hanan (2005 ▶). For similar dipyrazinyl­pyridine compounds, see: Dares *et al.* (2011 ▶); Dai *et al.* (2010*a* ▶,*b* ▶); Vougioukalakis *et al.* (2010 ▶); Liegghio *et al.* (2001 ▶).
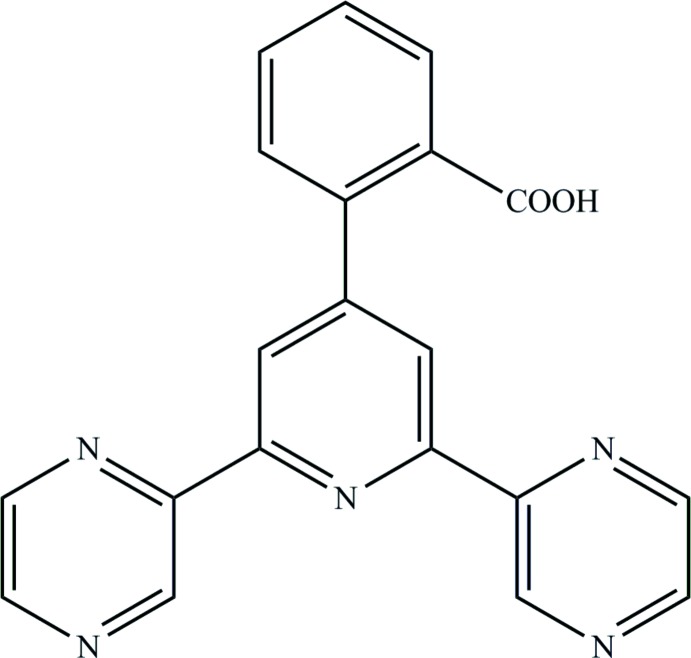


## Experimental   

### 

#### Crystal data  

C_20_H_13_N_5_O_2_
*M* *_r_* = 355.35
Triclinic, 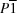

*a* = 7.0253 (9) Å
*b* = 10.9070 (14) Å
*c* = 11.3218 (14) Å
α = 99.345 (2)°
β = 99.266 (2)°
γ = 102.513 (2)°
*V* = 818.17 (18) Å^3^
*Z* = 2
Mo *K*α radiation
μ = 0.10 mm^−1^
*T* = 298 K
0.22 × 0.16 × 0.15 mm

#### Data collection  

Bruker APEXII CCD diffractometer
Absorption correction: multi-scan (*SADABS*; Bruker, 2002 ▶) *T*_min_ = 0.979, *T*_max_ = 0.985
4395 measured reflections
2998 independent reflections
1732 reflections with *I* > 2σ(*I*)
*R*_int_ = 0.022

#### Refinement  

*R*[*F* ^2^ > 2σ(*F* ^2^)] = 0.049
*wR*(*F* ^2^) = 0.137
*S* = 1.00
2998 reflections
244 parameters
H-atom parameters constrained
Δρ_max_ = 0.16 e Å^−3^
Δρ_min_ = −0.17 e Å^−3^

### 

Data collection: *APEX2* (Bruker, 2004 ▶); cell refinement: *SAINT* (Bruker, 2004 ▶); data reduction: *SAINT*; program(s) used to solve structure: *SHELXS97* (Sheldrick, 2008 ▶); program(s) used to refine structure: *SHELXL97* (Sheldrick, 2008 ▶); molecular graphics: *ORTEP-3 for Windows* (Farrugia, 2012 ▶); software used to prepare material for publication: *PLATON* (Spek, 2009 ▶).

## Supplementary Material

Crystal structure: contains datablock(s) I, global. DOI: 10.1107/S1600536814008496/mw2121sup1.cif

Structure factors: contains datablock(s) I. DOI: 10.1107/S1600536814008496/mw2121Isup2.hkl

Supporting information file. DOI: 10.1107/S1600536814008496/mw2121Isup3.cdx

Supporting information file. DOI: 10.1107/S1600536814008496/mw2121Isup4.cml

CCDC reference: 997338

Additional supporting information:  crystallographic information; 3D view; checkCIF report

## Figures and Tables

**Table 1:** Hydrogen-bond geometry (Å, °)

*D*—H⋯*A*	*D*—H	H⋯*A*	*D*⋯*A*	*D*—H⋯*A*
O1—H1*A*⋯O2^i^	0.82	1.84	2.656 (3)	176
C15—H15⋯N2^ii^	0.93	2.56	3.438 (3)	157

Symmetry codes: (i) 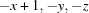; (ii) 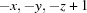.

**Fig. 1.**
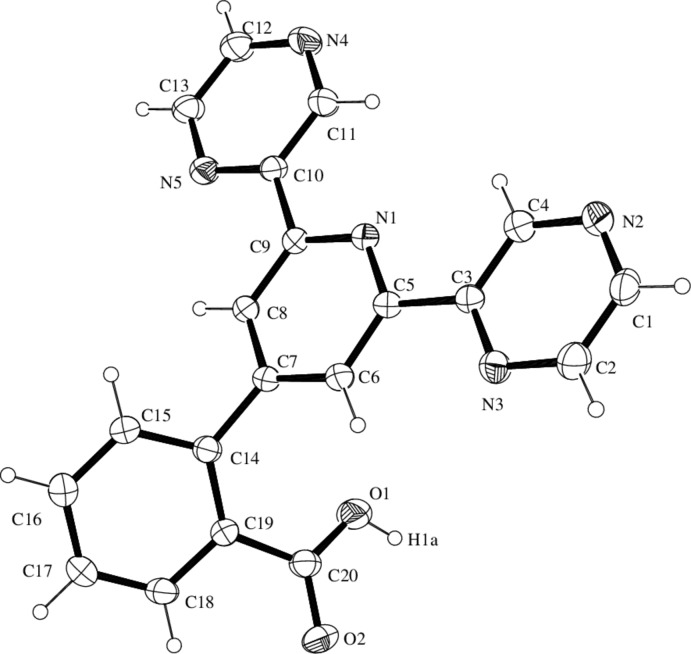
Molecular ORTEP diagram of (I) with atomic numbering. Displacement ellipsoids are drawn at the 50% probability and H atoms are shown as small spheres of arbitrary radii.

**Fig. 2.**
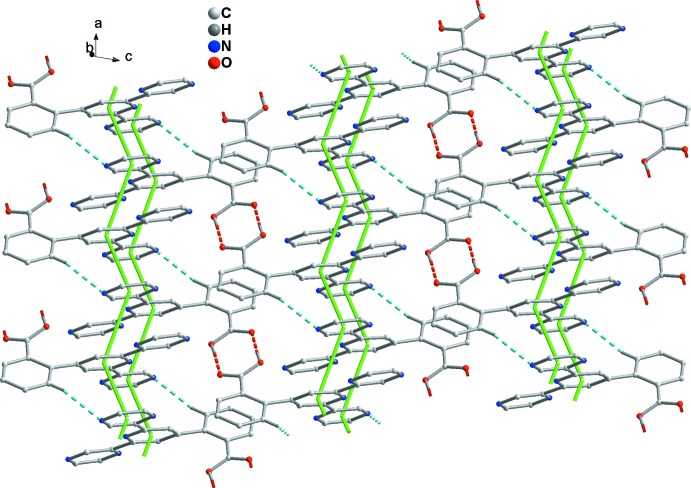
Crystal packing of (I). The aromatic stacking interactions are shown as solid green lines. The O–H···O and C–H···N hydrogen bonds are shown as dashed red and light blue lines, respectively. The H atoms not involved with the hydrogen bonds are omitted for clarity.

## supplementary crystallographic information

### 1. Comment

Terpyridine compounds are among the most frequently used tridentate ligands for transition metal elements (Constable, 2008; Eryazici *et al.*, 2008; Schubert *et al.*, 2006; Wild *et al.*). However, the similar dipyrazinylpyridine compounds have received much less attention and only a few structures have been reported (Dares *et al.*, 2011; Dai *et al.*, 2010*a*; Dai *et al.*, 2010*b*; Vougioukalakis *et al.*, 2010; Liegghio *et al.*, 2001). We previously demonstrated that 4-*p*-tolyl-2,6-di(pyrazin-2-yl)pyridine has stronger intermolecular interactions than its isoelectronic counterpart 4-*p*-tolyl-2,2':6',2''-terpyridine (suggested by a 90°C higher melting point for the former) and their coordination behaviours are also somewhat different (Dai *et al.*, 2010*a*). Herein we report the synthesis of 4-*o*-carboxyphenyl-2,6-di(pyrazin-2-yl)pyridine, (I), the first carboxyl-containing dipyrazinylpyridine compound, via by the Kröhnke reaction in a very convenient one-pot method in quantitative yield which contrasts with the frequently used two-step procedures for preparation of many terpyridine compounds (Schubert *et al.*, 2006). The molecular structure of (I) is shown in Fig. 1. Both pyrazinyl rings have their *ortho* N-atoms *anti* to the central pyridyl N atom. This conformation is free of possible steric strain between the vicinal C–H groups of the central pyridyl and the peripheral pyrazinyl rings and also avoids the lone-pair repulsions between *syn-*- positioned N atoms (Zadykowicz & Potvin, 1999) as is commonly found for the non-coordinated terpyridine or dipyrazinylpyridine compounds (Dares *et al.*, 2011; Dai *et al.*, 2010*b*; Vougioukalakis *et al.*, 2010; Liegghio *et al.*, 2001). The two pyrazinyl rings are nearly coplanar with the pyridyl ring as the dihedral angles between the N3- and N5-containing pyrazinyl rings and the pyridyl ring are 2.21 (9)° and 4.57 (9)°, respectively. In contrast, the strong steric hindrance caused by the *ortho*-carboxyl group on the phenyl ring makes this ring rotate out of the attached pyridyl plane by 52.60 (9)°. The carboxyl group twists from the phenyl ring by 22.6 (1)°.

The O–H···O and C–H···N hydrogen bonds (Table 1) as well as the aromatic π–π stacking interactions direct the crystal packing. As displayed in Fig. 2, each planar dipyrazinylpyridine moiety is antiparallel to two other moieties above and below it generating π–π stacking interactions as evidenced by the centroid-centroid distances between the nearly parallel aromatic rings: *Cg*(N2/N3/C1–C4)···*Cg*(N1/C5–C9)^i^ 3.9186 (4) Å and *Cg*(N2/N3/C1–C4)···*Cg*(N1/C5–C9)^ii^ 3.9794 (5) Å [symmetry codes: (i) –*x*, –*y*, –*z+*1; (ii) *x*+1, *y*, *z*]. The presence of a C15–H15···N2^i^ hydrogen bond (Table 1, Fig. 2) may account for the fact that the former centroid-centroid distance is a little shorter than the latter. The continuous stacking of the pyridyl and the N2-containing pyrazinyl rings leads to formation of infinite one-dimensional chains along the (100) direction. Between the neighbouring chains there are strong O1–H1*A*···O2^iii^ [(iii) –*x*+1, –*y*, –*z*] hydrogen bonds (Table 1, Fig. 2) forming a two-dimensional supramolecular network parallel to (010). There are no significant interactions among these sheets.

### 2. Experimental

To 15 mL of a methanolic solution of 2-acetylpyrazine (0.813 g, 6.7 mmol) and 2-carboxybenzaldehyde (0.5 g, 3.3 mmol) was added 10 mL of an aqueous solution of potassium hydroxide (3.57 mol·L^–1^) and then 15 mL of concentrated ammonia. After stirring for 24 h, the solution was acidified to pH 3–4 using hydrochloric acid. The resulting yellow precipitate was collected and washed with water and ethanol (yield 97%). Yellow crystals were obtained by recrystallization from chloroform, m.p. 282.2–284.0°C. IR (υ/cm^–1^): 3134, 3047, 1717, 1604, 1470, 1374, 1250, 1119, 1015, 850, 760, 690, 626, 480; ^1^H NMR (400 MHz, CDCl_3,_ TMS, *δ*/ppm): 9.83 (s, 2H, pyrazinyl NC*H*CN), 8.61 (m, 4H, pyrazinyl NC*H*C*H*N), 8.46 (s, 2H, pyridyl NCC*H*), 8.06 (d, 1H, phenyl C*H*CCOOH), 7.59 (t, 1H, phenyl C*H*CHCHCCOOH), 7.51 (t, 1H, phenyl C*H*CHCHCHCCOOH), 7.43 (d, 1H, phenyl C*H*CHCCOOH); ESI-MS: m/z = 354 ([M–H]^–^).

### 3. Refinement

H atoms attached to C atoms were positioned geometrically and allowed to ride on their parent atoms, with C–H = 0.93 Å and *U*_iso_(H) = 1.2*U*_eq_(C). The carboxyl H atom was located in a difference Fourier map and treated with the riding-model approximation with *U*_iso_(H) = 1.5*U*_eq_(O).

### Crystal data

**Table:**

C_20_H_13_N_5_O_2_	*Z* = 2
*M**_r_* = 355.35	*F*(000) = 368
Triclinic, *P*1	*D*_x_ = 1.442 Mg m^−^^3^
Hall symbol: -P 1	Mo *K*α radiation, λ = 0.71073 Å
*a* = 7.0253 (9) Å	Cell parameters from 753 reflections
*b* = 10.9070 (14) Å	θ = 3.2–23.9°
*c* = 11.3218 (14) Å	µ = 0.10 mm^−^^1^
α = 99.345 (2)°	*T* = 298 K
β = 99.266 (2)°	Block, yellow
γ = 102.513 (2)°	0.22 × 0.16 × 0.15 mm
*V* = 818.17 (18) Å^3^	

### Data collection

**Table:**

Bruker APEXII CCD diffractometer	2998 independent reflections
Radiation source: fine-focus sealed tube	1732 reflections with *I* > 2σ(*I*)
Graphite monochromator	*R*_int_ = 0.022
phi and ω scans	θ_max_ = 25.5°, θ_min_ = 1.9°
Absorption correction: multi-scan (*SADABS*; Bruker, 2002)	*h* = −8→7
*T*_min_ = 0.979, *T*_max_ = 0.985	*k* = −12→13
4395 measured reflections	*l* = −13→11

### Refinement

**Table:**

Refinement on *F*^2^	Primary atom site location: structure-invariant direct methods
Least-squares matrix: full	Secondary atom site location: difference Fourier map
*R*[*F*^2^ > 2σ(*F*^2^)] = 0.049	Hydrogen site location: inferred from neighbouring sites
*wR*(*F*^2^) = 0.137	H-atom parameters constrained
*S* = 1.00	*w* = 1/[σ^2^(*F*_o_^2^) + (0.0595*P*)^2^ + 0.0149*P*] where *P* = (*F*_o_^2^ + 2*F*_c_^2^)/3
2998 reflections	(Δ/σ)_max_ < 0.001
244 parameters	Δρ_max_ = 0.16 e Å^−^^3^
0 restraints	Δρ_min_ = −0.17 e Å^−^^3^

### Special details

**Table:**

Geometry. All esds (except the esd in the dihedral angle between two l.s. planes) are estimated using the full covariance matrix. The cell esds are taken into account individually in the estimation of esds in distances, angles and torsion angles; correlations between esds in cell parameters are only used when they are defined by crystal symmetry. An approximate (isotropic) treatment of cell esds is used for estimating esds involving l.s. planes.
Refinement. Refinement of *F*^2^ against ALL reflections. The weighted *R*-factor *wR* and goodness of fit *S* are based on *F*^2^, conventional *R*-factors *R* are based on *F*, with *F* set to zero for negative *F*^2^. The threshold expression of *F*^2^ > σ(*F*^2^) is used only for calculating *R*-factors(gt) *etc*. and is not relevant to the choice of reflections for refinement. *R*-factors based on *F*^2^ are statistically about twice as large as those based on *F*, and *R*- factors based on ALL data will be even larger.

### Fractional atomic coordinates and isotropic or equivalent isotropic displacement parameters (Å^2^)

**Table:**

	*x*	*y*	*z*	*U*_iso_*/*U*_eq_	
C1	0.1728 (4)	−0.3687 (3)	0.4584 (3)	0.0532 (7)	
H1	0.1637	−0.4541	0.4627	0.064*	
C2	0.1161 (4)	−0.3393 (3)	0.3473 (3)	0.0557 (8)	
H2	0.0671	−0.4056	0.2790	0.067*	
C3	0.1994 (3)	−0.1281 (2)	0.4341 (2)	0.0380 (6)	
C4	0.2524 (4)	−0.1594 (3)	0.5464 (2)	0.0495 (7)	
H4	0.2986	−0.0936	0.6153	0.059*	
C5	0.2180 (3)	0.0075 (2)	0.4215 (2)	0.0367 (6)	
C6	0.1703 (4)	0.0386 (2)	0.3081 (2)	0.0402 (6)	
H6	0.1276	−0.0256	0.2378	0.048*	
C7	0.1866 (4)	0.1655 (2)	0.29993 (19)	0.0366 (6)	
C8	0.2548 (3)	0.2569 (2)	0.4075 (2)	0.0385 (6)	
H8	0.2702	0.3434	0.4059	0.046*	
C9	0.3002 (3)	0.2190 (2)	0.5181 (2)	0.0359 (6)	
C10	0.3715 (3)	0.3160 (2)	0.63435 (19)	0.0361 (6)	
C11	0.4319 (4)	0.2822 (3)	0.7455 (2)	0.0487 (7)	
H11	0.4242	0.1963	0.7470	0.058*	
C12	0.5066 (4)	0.4901 (3)	0.8416 (2)	0.0560 (8)	
H12	0.5546	0.5539	0.9120	0.067*	
C13	0.4448 (4)	0.5244 (3)	0.7330 (2)	0.0508 (7)	
H13	0.4507	0.6102	0.7324	0.061*	
C14	0.1170 (4)	0.2064 (2)	0.1841 (2)	0.0387 (6)	
C15	−0.0200 (4)	0.2817 (3)	0.1901 (2)	0.0500 (7)	
H15	−0.0553	0.3058	0.2646	0.060*	
C16	−0.1048 (4)	0.3215 (3)	0.0891 (2)	0.0619 (8)	
H16	−0.1973	0.3704	0.0960	0.074*	
C17	−0.0530 (4)	0.2893 (3)	−0.0211 (2)	0.0583 (8)	
H17	−0.1086	0.3168	−0.0891	0.070*	
C18	0.0815 (4)	0.2162 (2)	−0.0302 (2)	0.0472 (7)	
H18	0.1162	0.1944	−0.1053	0.057*	
C19	0.1680 (4)	0.1736 (2)	0.0699 (2)	0.0384 (6)	
C20	0.3147 (4)	0.0977 (2)	0.0456 (2)	0.0441 (7)	
N1	0.2823 (3)	0.09612 (19)	0.52578 (16)	0.0382 (5)	
N2	0.2399 (4)	−0.2799 (2)	0.5597 (2)	0.0571 (7)	
N3	0.1286 (3)	−0.2189 (2)	0.33310 (18)	0.0492 (6)	
N4	0.5000 (4)	0.3688 (2)	0.84957 (18)	0.0592 (7)	
N5	0.3768 (3)	0.4380 (2)	0.62871 (17)	0.0457 (6)	
O1	0.4479 (3)	0.0935 (2)	0.13504 (16)	0.0668 (6)	
H1A	0.5194	0.0491	0.1102	0.100*	
O2	0.3067 (3)	0.0435 (2)	−0.06218 (15)	0.0668 (6)	

### Atomic displacement parameters (Å^2^)

**Table:**

	*U*^11^	*U*^22^	*U*^33^	*U*^12^	*U*^13^	*U*^23^
C1	0.067 (2)	0.0413 (17)	0.0645 (19)	0.0228 (14)	0.0308 (16)	0.0198 (15)
C2	0.071 (2)	0.0374 (17)	0.0585 (18)	0.0121 (15)	0.0171 (15)	0.0081 (14)
C3	0.0415 (15)	0.0388 (15)	0.0364 (14)	0.0119 (12)	0.0116 (11)	0.0094 (11)
C4	0.0631 (19)	0.0448 (17)	0.0455 (16)	0.0180 (14)	0.0160 (14)	0.0120 (13)
C5	0.0412 (14)	0.0390 (15)	0.0321 (13)	0.0125 (11)	0.0096 (11)	0.0080 (11)
C6	0.0514 (16)	0.0365 (15)	0.0327 (13)	0.0140 (12)	0.0070 (11)	0.0052 (11)
C7	0.0460 (15)	0.0370 (15)	0.0301 (13)	0.0138 (12)	0.0103 (11)	0.0088 (11)
C8	0.0513 (16)	0.0356 (15)	0.0345 (13)	0.0166 (12)	0.0136 (12)	0.0115 (11)
C9	0.0400 (14)	0.0372 (15)	0.0347 (13)	0.0149 (11)	0.0113 (11)	0.0088 (11)
C10	0.0382 (14)	0.0396 (15)	0.0318 (13)	0.0104 (11)	0.0099 (11)	0.0066 (11)
C11	0.0630 (18)	0.0461 (17)	0.0367 (14)	0.0169 (14)	0.0069 (13)	0.0068 (12)
C12	0.070 (2)	0.0503 (19)	0.0410 (16)	0.0121 (15)	0.0063 (14)	−0.0011 (13)
C13	0.071 (2)	0.0388 (16)	0.0417 (15)	0.0114 (14)	0.0150 (14)	0.0039 (12)
C14	0.0488 (16)	0.0344 (14)	0.0326 (13)	0.0099 (12)	0.0071 (11)	0.0072 (11)
C15	0.0677 (19)	0.0558 (18)	0.0362 (15)	0.0306 (15)	0.0163 (13)	0.0107 (13)
C16	0.077 (2)	0.075 (2)	0.0508 (17)	0.0470 (18)	0.0171 (16)	0.0203 (15)
C17	0.078 (2)	0.068 (2)	0.0402 (16)	0.0362 (17)	0.0102 (15)	0.0206 (14)
C18	0.0608 (18)	0.0507 (18)	0.0310 (14)	0.0151 (14)	0.0101 (12)	0.0079 (12)
C19	0.0446 (15)	0.0363 (15)	0.0339 (13)	0.0122 (12)	0.0070 (11)	0.0044 (11)
C20	0.0526 (17)	0.0422 (16)	0.0350 (14)	0.0090 (13)	0.0080 (13)	0.0051 (12)
N1	0.0465 (13)	0.0385 (13)	0.0334 (11)	0.0149 (10)	0.0114 (9)	0.0091 (9)
N2	0.0798 (18)	0.0509 (16)	0.0544 (15)	0.0276 (13)	0.0260 (13)	0.0226 (12)
N3	0.0692 (16)	0.0344 (13)	0.0439 (13)	0.0128 (11)	0.0098 (11)	0.0095 (10)
N4	0.0834 (18)	0.0574 (17)	0.0339 (12)	0.0208 (14)	0.0025 (12)	0.0060 (11)
N5	0.0593 (15)	0.0392 (13)	0.0392 (12)	0.0137 (11)	0.0111 (11)	0.0065 (10)
O1	0.0674 (14)	0.0972 (17)	0.0447 (11)	0.0458 (12)	0.0097 (10)	0.0071 (11)
O2	0.0819 (15)	0.0797 (15)	0.0412 (11)	0.0400 (12)	0.0104 (10)	−0.0052 (10)

### Geometric parameters (Å, º)

**Table:**

C1—N2	1.320 (3)	C11—N4	1.331 (3)
C1—C2	1.367 (3)	C11—H11	0.9300
C1—H1	0.9300	C12—N4	1.332 (3)
C2—N3	1.334 (3)	C12—C13	1.373 (3)
C2—H2	0.9300	C12—H12	0.9300
C3—N3	1.331 (3)	C13—N5	1.331 (3)
C3—C4	1.384 (3)	C13—H13	0.9300
C3—C5	1.488 (3)	C14—C15	1.397 (3)
C4—N2	1.334 (3)	C14—C19	1.408 (3)
C4—H4	0.9300	C15—C16	1.380 (3)
C5—N1	1.342 (3)	C15—H15	0.9300
C5—C6	1.389 (3)	C16—C17	1.369 (3)
C6—C7	1.382 (3)	C16—H16	0.9300
C6—H6	0.9300	C17—C18	1.367 (4)
C7—C8	1.385 (3)	C17—H17	0.9300
C7—C14	1.496 (3)	C18—C19	1.394 (3)
C8—C9	1.391 (3)	C18—H18	0.9300
C8—H8	0.9300	C19—C20	1.486 (3)
C9—N1	1.337 (3)	C20—O2	1.254 (3)
C9—C10	1.486 (3)	C20—O1	1.277 (3)
C10—N5	1.336 (3)	O1—H1A	0.8200
C10—C11	1.395 (3)		
			
N2—C1—C2	122.2 (3)	N4—C12—C13	122.3 (2)
N2—C1—H1	118.9	N4—C12—H12	118.8
C2—C1—H1	118.9	C13—C12—H12	118.8
N3—C2—C1	122.5 (3)	N5—C13—C12	121.8 (3)
N3—C2—H2	118.8	N5—C13—H13	119.1
C1—C2—H2	118.8	C12—C13—H13	119.1
N3—C3—C4	120.9 (2)	C15—C14—C19	117.0 (2)
N3—C3—C5	117.6 (2)	C15—C14—C7	115.5 (2)
C4—C3—C5	121.6 (2)	C19—C14—C7	127.4 (2)
N2—C4—C3	122.7 (3)	C16—C15—C14	122.1 (2)
N2—C4—H4	118.7	C16—C15—H15	118.9
C3—C4—H4	118.7	C14—C15—H15	118.9
N1—C5—C6	122.8 (2)	C17—C16—C15	120.1 (3)
N1—C5—C3	115.9 (2)	C17—C16—H16	119.9
C6—C5—C3	121.3 (2)	C15—C16—H16	119.9
C7—C6—C5	119.8 (2)	C18—C17—C16	119.3 (3)
C7—C6—H6	120.1	C18—C17—H17	120.3
C5—C6—H6	120.1	C16—C17—H17	120.3
C6—C7—C8	117.3 (2)	C17—C18—C19	121.8 (2)
C6—C7—C14	123.3 (2)	C17—C18—H18	119.1
C8—C7—C14	119.1 (2)	C19—C18—H18	119.1
C7—C8—C9	119.9 (2)	C18—C19—C14	119.6 (2)
C7—C8—H8	120.1	C18—C19—C20	115.3 (2)
C9—C8—H8	120.1	C14—C19—C20	125.2 (2)
N1—C9—C8	122.6 (2)	O2—C20—O1	122.5 (3)
N1—C9—C10	117.0 (2)	O2—C20—C19	118.8 (2)
C8—C9—C10	120.4 (2)	O1—C20—C19	118.7 (2)
N5—C10—C11	120.8 (2)	C9—N1—C5	117.6 (2)
N5—C10—C9	117.4 (2)	C1—N2—C4	115.6 (2)
C11—C10—C9	121.8 (2)	C3—N3—C2	116.1 (2)
N4—C11—C10	122.2 (3)	C11—N4—C12	116.1 (2)
N4—C11—H11	118.9	C13—N5—C10	116.8 (2)
C10—C11—H11	118.9	C20—O1—H1A	109.5
			
N2—C1—C2—N3	1.4 (4)	C14—C15—C16—C17	−1.1 (4)
N3—C3—C4—N2	1.4 (4)	C15—C16—C17—C18	0.7 (5)
C5—C3—C4—N2	−179.0 (2)	C16—C17—C18—C19	−0.1 (4)
N3—C3—C5—N1	177.5 (2)	C17—C18—C19—C14	−0.2 (4)
C4—C3—C5—N1	−2.2 (3)	C17—C18—C19—C20	−178.6 (3)
N3—C3—C5—C6	−2.4 (3)	C15—C14—C19—C18	−0.1 (4)
C4—C3—C5—C6	178.0 (2)	C7—C14—C19—C18	177.1 (2)
N1—C5—C6—C7	−0.7 (4)	C15—C14—C19—C20	178.1 (2)
C3—C5—C6—C7	179.1 (2)	C7—C14—C19—C20	−4.6 (4)
C5—C6—C7—C8	1.2 (3)	C18—C19—C20—O2	−22.1 (3)
C5—C6—C7—C14	−172.8 (2)	C14—C19—C20—O2	159.6 (2)
C6—C7—C8—C9	−1.1 (3)	C18—C19—C20—O1	155.8 (2)
C14—C7—C8—C9	173.2 (2)	C14—C19—C20—O1	−22.5 (4)
C7—C8—C9—N1	0.5 (4)	C8—C9—N1—C5	0.1 (3)
C7—C8—C9—C10	−179.4 (2)	C10—C9—N1—C5	179.9 (2)
N1—C9—C10—N5	−175.5 (2)	C6—C5—N1—C9	0.0 (3)
C8—C9—C10—N5	4.4 (3)	C3—C5—N1—C9	−179.8 (2)
N1—C9—C10—C11	4.8 (3)	C2—C1—N2—C4	−1.2 (4)
C8—C9—C10—C11	−175.3 (2)	C3—C4—N2—C1	−0.2 (4)
N5—C10—C11—N4	−1.2 (4)	C4—C3—N3—C2	−1.2 (4)
C9—C10—C11—N4	178.5 (2)	C5—C3—N3—C2	179.2 (2)
N4—C12—C13—N5	−0.8 (4)	C1—C2—N3—C3	−0.2 (4)
C6—C7—C14—C15	123.9 (3)	C10—C11—N4—C12	0.3 (4)
C8—C7—C14—C15	−50.1 (3)	C13—C12—N4—C11	0.7 (4)
C6—C7—C14—C19	−53.4 (4)	C12—C13—N5—C10	−0.1 (4)
C8—C7—C14—C19	132.6 (3)	C11—C10—N5—C13	1.1 (3)
C19—C14—C15—C16	0.8 (4)	C9—C10—N5—C13	−178.6 (2)
C7—C14—C15—C16	−176.8 (3)		

### Hydrogen-bond geometry (Å, º)

**Table:**

*D*—H···*A*	*D*—H	H···*A*	*D*···*A*	*D*—H···*A*
O1—H1*A*···O2^i^	0.82	1.84	2.656 (3)	176
C15—H15···N2^ii^	0.93	2.56	3.438 (3)	157

Symmetry codes: (i) −*x*+1, −*y*, −*z*; (ii) −*x*, −*y*, −*z*+1.
